# Editorial: Preclinical Animal Models and Measures of Pain: Improving Predictive Validity for Analgesic Drug Development

**DOI:** 10.3389/fpain.2022.867786

**Published:** 2022-03-23

**Authors:** Anke Tappe-Theodor, S. Stevens Negus, Thomas J. Martin

**Affiliations:** ^1^Institute of Pharmacology, University of Heidelberg, Heidelberg, Germany; ^2^Department of Pharmacology and Toxicology, Virginia Commonwealth University, Richmond, VA, United States; ^3^Pain Mechanisms Laboratory, Department of Anesthesiology, Wake Forest University School of Medicine, Winston-Salem, NC, United States

**Keywords:** pain, animal model, analgesics, drug development, inflammation, neuropathic, chemotherapy induced neuropathic pain, surgical pain

This special issue highlights studies that address current limitations in the methods for preclinical development of analgesics and presents novel approaches and concepts providing insight into pathways for improved translation from bench to bedside. The lack of progress in translational efforts for novel analgesic development has been highlighted in the literature for some time (1–3). However, the sophistication in research design and methods that has evolved over the last decades has been impressive and noteworthy. Decades ago, preclinical research on pain mechanisms primarily relied on stimulation of spinal reflex arcs with acute noxious stimuli and measurement of response latency of reflexive withdrawal of the paw or tail. Over several decades, novel pain models evolved to mimic clinical pathological states related to pain more closely. However, development of behavioral endpoints related to pain lagged behind these efforts. Reflexive withdrawal from noxious stimuli continued to serve as the primary endpoint used to indicate efficacy for candidate analgesics. After decades of such methods failing to provide predictive value for advancing novel pain therapeutics into the clinic, the need for behavioral sophistication became apparent and prioritized.

Pain is a multidimensional experience with sensory/discriminative, affective/motivational, and cognitive aspects (4, 5). While early behavioral measures included primarily sensory/discriminative aspects, as noted above, advances have been made in devising endpoints that assess the affective/motivational and cognitive dimensions (6–9). However, many behavioral assays are not as sensitive to pain manipulations as the classical reflexive withdrawal endpoints. Moreover, several paradigms require extensive behavioral training, specialized and costly equipment, and in general, may lack sufficient throughput to assess potential efficacy of candidate compounds in an efficient and cost-effective manner. Nonetheless, such methods must continue to be developed and explored, both for evaluating novel analgesics and providing means to assess the relevance of novel targets for pain treatment using biochemical and molecular tools such as RNAseq, genetic manipulations, chemogenetic or optogenetic methods, among others.

Several factors are critical in evaluating novel pain models and behavioral methods and endpoints, and in this issue, we highlight three aspects. The first is face validity. Pain is most problematic when associated with pathological conditions, and new animal models should ideally recapitulate both the underlying pathology and the behavioral symptomology as closely as possible. Second is the ability to distinguish between the effects of positive and negative controls. New preclinical methods should reduce the vulnerability to false-positive results by preserving positive responses to analgesics with known effectiveness (positive controls) while yielding negative results with treatments known to lack effectiveness (negative controls). Although several clinically relevant pain conditions lack highly effective therapies, it is useful to examine partially effective positive controls and pertinent negative controls in preclinical models of such conditions. A final challenge in model development is the potential for negative or neutral outcomes of development or replication efforts to influence publication. Publication of negative or neutral data however provides the field at large with critical information that can be used to refine future model development or give insight into the reproducibility of current models across laboratories. With this in mind, several articles in this issue provide evidence for well-designed studies that tested but did not support a rational hypothesis. In our view, publication of both positive and negative results is critical to guide future model-development efforts.

The studies in this special issue utilized both longstanding and novel approaches to induce various pathological pain states. These range from classical methods to induce inflammation or neuropathy to less studied models of bladder and intestinal pain, as well as novel approaches to develop models of phantom limb pain or headache. All studies utilized mice or rats, some compared the behavioral effects across more than one pathology, and various models with face validity for clinical pain and numerous behavioral methods were used to assess pain-stimulated or pain-depressed behavioral endpoints; mechanical or thermal hypersensitivity measures, effects on complex behaviors that include appetitive reward, nest building, exploratory behaviors, gait and posture, anxiety- and depression-related behaviors, and self-injurious behavior.

Auge et al., Cheatham et al., and Garner et al. examined bladder, colitis or diffuse abdominal inflammatory pain, respectively. Auge et al. demonstrated potential for T-cell stimulation to mitigate abdominal hypersensitivity, Cheatham et al. showed a lack of effect of ketoprofen or morphine in an irritable bowel syndrome model, and Garner et al. found that the efficacy of morphine against abdominal pain was dependent upon the strength of noxious input. Draxler et al. examined different pain models combined with standard and novel behavioral assessments and analgesics in rats. Their findings indicate that each pain model displays distinct sensitivities across behavioral measures, with the classical reflexive hypersensitivity measures being the most sensitive. Segelcke et al. and Salcido et al. compared the effects of paw inflammation with paw incision or nerve injury, respectively, on a variety of behaviors. Both manuscripts demonstrate varying effects of mechanical hypersensitivity of affected hindpaws on non-evoked or movement-evoked behaviors (Segelcke et al.) and no impact of noxious mechanical input on food-reinforced responding (Salcido et al.). Segelcke et al. further tested the idea that accumulation of neutrophil granulocytes altered complex behavioral responses to inflammation or incision. Jergova et al. developed a model of phantom limb pain, demonstrating that pre-existing inflammatory or neuropathic pain alters self-injurious behavior and that autotomy is directed to the site of previous hypersensitivity. Warncke et al., examined several classical reflexive and non-reflexive behaviors in male and female mice following oxaliplatin-induced neuropathy, finding that behavioral outcomes displayed different time courses and sensitivities to male vs. female mice, with classical reflexive withdrawal measures being most sensitive.

These studies collectively build upon the available tools to study select pain-related pathologies and behavioral outcomes, displaying positive and negative findings. Each method and model relates valuable data and conceptual insight toward the continued efforts to improve preclinical modeling of pain-related pathological states and behavioral assessments. The ultimate goal is to provide standardized methods to discover more efficacious pain treatments, and we are convinced that this special issue constitutes an important step toward this direction.

## Author Contributions

AT-T, SN, and TM wrote and edited the manuscript and approved submission. All authors contributed to the article and approved the submitted version.

## Funding

The authors acknowledge funding to AT-T from the Deutsche Forschungsgemeinschaft in the form of a Collaborative Research Centre 1158 grant (projects S01).

## Conflict of Interest

The authors declare that the research was conducted in the absence of any commercial or financial relationships that could be construed as a potential conflict of interest.

## Publisher's Note

All claims expressed in this article are solely those of the authors and do not necessarily represent those of their affiliated organizations, or those of the publisher, the editors and the reviewers. Any product that may be evaluated in this article, or claim that may be made by its manufacturer, is not guaranteed or endorsed by the publisher.
